# Understanding Group and Leader (UGL) trainers' personality characteristics and affective profiles

**DOI:** 10.3389/fpsyg.2014.01191

**Published:** 2014-10-21

**Authors:** Max Rapp Ricciardi, Jeanette Åkerman, Peter Eerikäinen, Annika Ambjörnsson, Ann-Christine Andersson Arntén, Marko Mihailovic, Trevor Archer, Danilo Garcia

**Affiliations:** ^1^Department of Psychology, University of GothenburgGothenburg, Sweden; ^2^Network for Empowerment and Well-BeingGothenburg, Sweden; ^3^Office of the National Police Commissioner, National Police BoardStockholm, Sweden; ^4^Institute of Neuroscience and Physiology, Centre for Ethics, Law and Mental Health, University of GothenburgGothenburg, Sweden

**Keywords:** affective profiles, coaching, leadership, locus of control, optimism, self-esteem, UGL

## Abstract

**Background:** The Understanding Group and Leader (UGL), provided by the Swedish National Defense College and mentored by UGL-trainers, is one of the most popular management programs among civilians in Sweden. However, there is a lack of scientific evidence regarding the training. We used the affective profile model (i.e., the combination of positive, PA, and negative affect, NA) to mapp important markers of empowerment, self-awareness, adaptive coping skills, and maturity among the UGL-trainers. The aims were: (1) to compare profiles between UGL-trainers and managers/supervisors and (2) to investigate differences in personal characteristics.

**Method:** UGL-trainers (*N* = 153) and the comparison group (104 Swedish Chiefs of Police) completed an online survey on optimism, self-esteem, locus of control, and affect. The four profiles are: self-fulfilling (high PA, low NA), high affective (high PA, high NA), low affective (high PA, low NA), and self-destructive (low PA, high NA).

**Results:** The self-fulfilling profile was more common among UGL-trainers (25.70%) than among Chiefs of Police (19.20%). UGL-trainers, compared to Chiefs of Police, were more likely to express a self-fulling than a low affective profile (OR = 2.22, *p* < 0.05) and a high affective than a low affective profile (OR = 1.43, *p* < 0.001). UGL-trainers with a self-fulfilling profile, compared to those with a self-destructive profile, scored higher in optimism, higher in self-esteem, and lower in external locus of control.

**Conclusions:** The probability of self-fulfillment rather than low affectivity was higher among UGL-trainers. Self-fulfillment was associated to markers of self-awareness and adaptive coping skills. However, the most common profile was the low affective, which is associated to low performance during stress, low degree of personal development, low degree of purpose in life, and low resilience. Hence, it might be important for UGL-trainers to have a continuous training in awareness after certification.

## Introduction

UGL is an acronym of the Swedish words: “Utveckling Grupp Ledare” which may be translated as: Understanding Group and Leader. The UGL is one of the most popular management training programs in Sweden and it is estimated that approximately 4000 individuals/year participate in the program (Rapp Ricciardi and Räisänen, unpublished). Provided by the Swedish National Defense College (SNDC), it was introduced in Sweden in 1981 and was initially planned as a type of leadership training only for officers and cadets in the Swedish Armed Forces, but it soon gained popularity in civilian contexts as well (SNDC, 2014a). Professional certified UGL-trainers work in pairs to coach the participants through the training days of the program. Criticism against the training revolves around claims that the group processes, on which the program is based on, require more skills from the UGL-trainers than it may be provided during the 5-week (1 + 2 + 2 weeks) the trainers need to go through in order to gain certification (e.g., Fellinger, 2012a,b,c; SNDC, 2013). Nevertheless, the course has enjoyed great approval in Swedish work-life and is commonly believed to influence occupational leadership and is popular among organizations in both the private and public sector. The SNDC owns copyright of the concept and certify the trainers, some of whom are employed by the SNDC and some are management consultants using the concept under licensing restrictions. Currently, the SNDC has initiated a quality control process with the purpose to increase transparency by allowing researchers to analyze various aspects of the program (SNDC, 2013). This study is part of this initiative and focuses on mapping personal characteristics of the certified UGL-trainers.

The UGL basic training course is designed as follows: There must be at least 8 but not more than 12 participants during the 5-day long course. All of the participants are expected to be complete strangers to one another at the start of the course (SNDC, 2014b), since exploration of interpersonal relationships is a major aspect of the learning climate during the course (i.e., experienced-based learning; Kolb, 1984). The participants are exposed to different exercises that aim to develop skills related to decision-making, perception, cognitive flexibility, and emotional control. These exercises are designed to generate cognitive and emotional conflicts of diverse nature. Participants are encouraged to express, communicate and provide feed-back about their observations and feelings when testing novel and more adequate approaches and behaviors that aim to improve the quality of their collaboration skills. The fundamental notion of the program is to provide a positive experience of a “muddling-through” process; which might end in mistrust and conflict but yet allows for the possibility to evolve into a process of mutual trust and cooperation by improvement in communication skills.

The basic theoretical structure of the UGL has evolved from Schutz (1958) FIRO-model that describes individuals' fundamental needs in certain phases of the life cycle of any group (i.e., inclusion, control, and affection). In 2008, the SNDC introduced the integrated model of group development into the UGL training, which is an evidence-based model with both research and a theoretical framework (Wheelan, 2010) stemming from group dynamics (Schutz, 1958; Bion, 1961; Tuckman and Jensen, 1977). In short, Wheelan's integrated model of group development (Wheelan, 2003, 2004; Wheelan et al., 2003) presents the notion of “stage-wise” group development: from the stage of “inclusion and dependence,” to the stage of “counterdependence and conflict,” and then to the stage of “trust and structure,” and finally the last stage of “work and productivity.” The feeling of “security” (i.e., stage 1 in Wheelan's model) is fundamental to the process of group maturation (Wheelan, 2010). This stage is characterized by the concerns of the group members regarding personal security with leader-dependency and need for structure and order. At this stage, the leader's role is essential since it implies both the “secure” climate and the necessary structure. Moreover, the social context influences individuals' expectations of their own behavior (Salancik and Pfeffer, 1978), in turn, dysfunctional behaviors influence the whole process (Felps et al., 2006). The group itself might cease to exist during any of these stages, with different consequences. According to Wheelan (1999) groups may provide either a positive or negative environment for the individual. In a group, individuals may, for example, experience a loss of identity and anonymity (Zimbardo, 1969, 2009), which might diminish the individuals' perception of autonomy (Wheelan, 1999). Bandura (1973) has suggested that members of a group may “imitate” dysfunctional behavior non-consciously through interpersonal observation. Unfortunately, individuals, exposed to dysfunctional behaviors are generally receptive to these behaviors (Felps et al., 2006), which lead to negative affectivity.

Affectivity is a personal attribute (Watson et al., 1988) describing how individuals perceive emotions in two dimensions: positive affect (PA) and negative affect (NA). PA is characterized by engagement, proudness, and attentiveness, while NA is characterized by fear, anger, guilt, and anxiety (Watson et al., 1988). Costa and McCrae (1980) have argued that individuals with a high degree of PA express also a high degree of self-esteem and a feeling of security/safety, whereas individuals expressing a high degree of NA report stress and anxiety in situations where they experience lack of control (Watson et al., 1986).

In relation to group dynamics, individuals expressing PA may influence the “affect balance” of the rest of the group thereby contributing positively to the social context (Hatfield et al., 1993). For instance, the “Broaden-and-build” model (Fredrickson, 2003) suggests that positive feelings may broaden the individuals' repertoire of thoughts and actions with consequential formation of personal and social resources. Individuals who experience high levels of PA are receptive to new information and attuned to discovery (Fredrickson, 2003). Consequently, these individuals are more willing to engage in new tasks and collaborative behavior (Fredrickson and Losada, 2005; Schütz et al., 2013) and also express greater creativity and problem-solving ability (Lyubomirsky et al., 2005). Their PA may infect to the other members of a group and catalyze functions within the group (Fredrickson, 2003). “Group-infection” may be particularly strong if a high status person, such as the leader, is the one who expresses high levels of PA (George, 1995; Quinn, 2000; Sy et al., 2005; Gooty et al., 2010). George and Bettenhausen (1990) showed, for example, that leaders experience of PA was positively related to the incidence of prosocial behavior among members of work groups and it was negatively related to employee turnover, which might lead to more effective conflict resolution (Barsade and Gibson, 2007). Contrastingly, if the leader expresses high levels of NA, groups perceive leader-feedback as less effective, which results in poor performance (Gaddis et al., 2004; Johnson, 2008; Gooty et al., 2010). Groups in which the leader experiences high levels of negative emotions tend to focus on the internal group relations, whereas those in which the group experiences high levels of positive emotions focus on the task (Grawitch et al., 2003).

### The affective profiles

Norlander et al. (2002) extended the notion of affectivity as two dimensions by combining the dimensions to four affective profiles: (i) “self-fulfilling” implying individuals high on PA and low on NA, (ii) “high-affective” with high PA and high NA, (iii) “low-affective” with low PA and low NA, and (iv) “self-destructive” with low PA and high NA. Garcia et al. (2014) focused on the correlation between the affective profiles and individuals' well-being and harmony in life and found that individuals with a self-fulfilling and/or high-affective profile, compared to low PA profiles (i.e., low affective and self-destructive), have and maintain positive relationships with others, are self-acceptant, have a sense of having control of their environment, experience a high degree of personal development, feel harmony in their life, and have a feeling of purpose in life. These two affective profiles, self-fulfilling and high-affective, were different only with regard to their sense of autonomy—individuals with a self-fulfilling profile experiencing higher level of autonomy compared to those with a high affective profile.

Moreover, Garcia et al. (2014) indicated that although individuals with a low-affective profile, compared to those with a self-destructive profile, also have good relationships, accept themselves, control their environment and experience harmony, they express a low degree of personal development and do not perceive that there is a purpose in their life. Nevertheless, individuals with a low affective profile perceive a high degree of autonomy, a feeling they share with individuals with a self-fulfilling profile. Importantly, individuals with a self-destructive profile expressed a low degree of all the factors mentioned above. The affective profiles have different levels of self-esteem, optimism, and locus of control (e.g., Archer et al., 2008). Individuals with a self-fulfilling profile express a high degree of optimism and internal locus of control, while persons with a self-destructive profile express low self-esteem, low degree of optimism, and an external locus of control (Archer et al., 2008). These three specific attributes (i.e., self-esteem, optimism, and locus of control) are markers of empowerment, self-awareness, adaptive coping skills, and maturity.

### Self-esteem, optimism, and locus of control

A high self-esteem implies that there is self-confidence and high trust in one's personal inner resources and strengths (Baumeister and Tice, 1985; Baumeister et al., 2001). Individuals with a high self-esteem and optimism have a tendency to perceive stressful situations as challenging rather than threatening (Kivimäki, 1996), thus feel more empowered in life. Optimism can be defined as the expectation of the realization of positive experiences through one's life (Scheier et al., 2001). Optimism equips individuals to deal with stressful situations more effectively, compared to individuals with a pessimistic worldview (Penedo et al., 2003). Furthermore, in contrast to pessimistic individuals, optimistic individuals have access to a greater variation of strategies to deal with stressful situations and good mental health (Scheier et al., 2001). In a meta-analysis involving 11,629 individuals, optimism was positively associated to approach coping strategies aimed at eliminating, reducing, or managing stressors or emotions, and negatively associated to avoidance coping strategies seeking to ignore, avoid, or withdraw from stressors and emotions (Nes and Segerstrom, 2006).

Individuals who have a sense of control over or expect themselves to be able to influence situations express an internal locus of control. In contrast, individuals who feel that they are controlled by the situations in their life express an external locus of control (Rotter, 1966). Individuals who experience internal locus of control are self-aware and mature (Cloninger and Garcia, 2014), hence, they use coping strategies that are more adaptive compared to those used by individuals who experience external locus of control (Parkes, 1984). Adeyemi-Bello (2003), for instance, showed that groups with leaders who reported having a high degree of internal locus of control performed more effectively (see also Johnson et al., 1984 who had earlier showed the linkt to performance but also to contentedness).

### The present study

In order to achieve the status of a certified UGL-trainer by the SNDC the candidate is required to pass through the following steps: 1 week of 5 days basic training (UGL), 2 weeks of 10 days advanced course (FUGL), 2 weeks of 10 days trainers' course (HUGL), and finally the candidate must run the course accompanied by a skilled co-trainer who assesses the candidates' skills (SNDC, 2014c). This process takes usually 2–3 years since it is recommended that the candidate has a period of reflection about her/himself between each of the different courses. In short, each course requires self-reflection, which is expected to lead to self-development, improving communicative skills and an increased understanding of group dynamics and interpersonal relationships.

The purpose of the present study was to examine the personal predispositions of the certified UGL-trainers. Specifically, the study aims (1) to compare the affective profiles of certified UGL-trainers to actual managers/supervisors of an organization and (2) to investigate personal characteristics of professional UGL-trainers in Sweden using the affective profiles as the framework for the investigation. The notion that affectivity is linked closely with self-esteem, optimism, locus of control, and that it also modulates leadership performance and perception is central in the present study, thereby the following hypotheses:

1. Since self-development is essential for the certified UGL-trainers, it was predicted that the probability of having a self-fulfilling profile would be higher among UGL-trainers than among the comparison group.
2. UGL-trainers with a self-fulfilling profile were expected to report higher levels of self-esteem, optimism, and locus of control than UGL-trainers with any of the other profiles, especially compared to UGL-trainers with a self-destructive profile.

## Methods

### Participants and procedure

The population was 416 active certified UGL-trainers, registered at the SNDC who provided their e-mail addresses in March 2014. A total of 153 completed the online survey (i.e., 63% dropout rate) that was accessible during a period of 4 weeks. The first reminder was sent to the participants after 2 weeks and a second reminder after 3 weeks. The survey took about 30–40 min to complete and it was technically possible to pause and complete it at a later occasion. In the instructions it was made clear that the study was a collaboration between SNDC and the University of Gothenburg, Sweden. The respondents were informed that all answers would be handled with total anonimity and used only for research and pedagogical purposes. Table 1 shows the distribution, across the UGL-sample, of gender, age, education, number of years since certification, number of courses per year, and number of courses lead by the trainer. According to law (2003: 460, §2) concerning the ethical research involving humans we arrived at the conclusion that the design of the present study (e.g., all participants' data were anonymous and will not be used for commercial or other non-scientific purposes) required only informed consent from participants.

**Table 1:** **UGL-trainers' demographics**.

**Gender**		**Number**	**Percent**	**No training I lead per/year**		**Number**	**Percent**
Valid	Man	75	49.0	Valid	0–5	124	81.0
	Woman	77	50.3		6–10	26	17.0
	Total	152	99.3		11–15	2	1.3
					Total	152	99.3
Missing		1	0.7	Missing		1	0.7
Total (Missing and valid)		153	100.00	Total (Missing and valid)		153	100.00
**Age**		**Number**	**Percent**	**No UGL I lead total**		**Number**	**Percent**
Valid	26–30	3	2.0	Valid	0–10	32	20.9
	31–35	3	2.0		11–20	24	15.7
	36–40	4	2.6		21–30	24	15.7
	41–45	16	10.5		31–40	12	7.8
	46–50	28	18.3		41–50	11	7.2
	51–55	28	18.3		51–60	10	6.5
	56–60	33	21.6		61–70	4	2.6
	61–64	14	9.2		71–80	2	1.3
	>65	24	15.7		81–90	4	2.6
	Total	153	100		91–100	5	3.3
		0	0.0		101–200	16	10.5
					201–300	4	2.6
					>300	2	1.3
					Total	150	98.0
Missing			100.00	Missing		3	2.0
Total		153	100.00	Total (valid and missing)		153	100.00
**Education/degree**		**Number**	**Percent**	**Holds other certifications (ICF, UL, THE, etc).**		**Number**	**Percent**
Valid	High school 2 yrs	6	3.9	Valid	Yes	120	78.4
	High school 3–4 yrs	16	10.5		No	32	20.9
	Folk high school	5	3.3		Total	152	99.3
	University B.Sc / M.Sc	118	77.1	Missing		1	7
	P.hd.	3	2.0	Total		153	100.00
	Vocational school	4	2.6				
	Total	152	99.3	**Active in other leadership/group dvp. Progs.**		Number	Percent
Missing		1	0.7	Valid	Yes	110	71.9
Total (valid and missing)		153	100.00		No	41	26.8
**Number of yrs as UGL trainer**		**Number**	**Percent**		Total	151	98.7
Valid	0–5	18	11.8	Missing		2	1.3
	6–10	35	22.9	Valid and missing		153	100.00
	11–15	40	26.1				
	16–20	26	17.0				
	21–25	19	12.4				
	26–30	10	6.5				
	>30	4	2.6				
	Total	152	99.3				
Missing		1	0.7				
Total (valid and missing)		153	100.0				

The comparison group consisted of 104 Swedish Chiefs of Police, 59 men and 45 women. This data was collected, also using an online survey, separetly for another study and is detailed somewhere else (Andersson Arntén et al., in press). While the UGL-trainers answered to all instruments detailed next, the only common measures with the comparison group was affectivity.

### Instruments

#### The positive affect and negative affect schedule (Watson et al., 1988)

This instrument measures PA and NA and consists of 20 adjectives, which describe different emotions and feelings. Ten adjectives describing PA (e.g., *“engaged,” “enthusiastic,” “proud,” “inspired”*) and 10 adjectives describing NA (e.g., *“frightened,” “ashamed,”* and *“nervous”*). The respondents are instructed to mark to what extent they perceived these during the last weeks in a 5-point Likert scale (1 = *very slightly*, 5 = *extremely*). Cronbach's alpha were 0.85 for PA and 0.82 for NA.

#### Life orientation test (Scheier and Carver, 1985)

This test measures optimism using 12 statements (e.g., *“In uncertain times I expect the best,” “I can relax easily”*) in a 5-point Likert scale (0 = *disagree completely* and 4 = *agree completely*). Cronbach's alpha was 0.67.

#### Rosenberg's self-esteem scale (Rosenberg, 1965)

The instrument consists of 10 statements (e.g.,*”On the whole I am satisfied with myself”*) measuring self-esteem in a 4-point Likert scale (1 = *Agree completely* and 4 = *Disagree completely*). Cronbach's alpha was 0.70.

#### Locus of control (Andersson, 1976)

This instrument measures to what extent an individual perceives internal and external locus of control and consists of 8 statements (e.g., *“I do not think there are such thing as luck or unluck which influences my life”*) using a 5-point Likert scale (1 = *Agree completely* and 5 = *Disagree completely*). Cronbach's alpha for internal locus of control was 0.59 and for external locus of control was 0.47.

### Statistical treatment

In order to categorize participants in different affective profiles, the data pertaining affectivity from the certified UGL-trainers and from the comparison group was merged with a larger sample and comprising about 1000 individuals from different professions, such white collar workers (for a more detailed description see Garcia et al., 2012; Moradi et al., 2014). A median split divided participants' PA and NA scores in high and low. Thereafter the high/low PA and NA categories were used to create the different profile combinations: self-fulfilling (high PA and low NA), low-affective (low PA and low NA), high-affective (high PA and high NA) and self-destructive (low PA and high NA). Archer and colleagues introduced this procedure in earlier studies (e.g., Norlander et al., 2002, 2005).

A *chi square* test was first used to investigate which profiles were more common among the Certified UGL-trainers compared to the Chiefs of Police. Furthermore, a Multinomial Logistic Regression (MLR) was used to see if being an Certified UGL-trainer, compared to being a Chief of Police, increased the probability of having a self-fulfilling affective profile. A Multivariate Analysis of Variance (MANOVA) was performed to test differences in optimism, self-esteem, and locus of control between affective profiles among UGL-trainers. A *post-hoc* test, with a Bonferroni correction of 0.0125 was used to investigate which profiles differed in the dependent variables (i.e., optimism, self-esteem, and locus of control). A Levene's test showed that the groups were not homogeneous for optimism and self-esteem, thus, the results were followed up with Kruskall-Wallis non-parametric test.

## Results

### Comparison between UGL-trainers and chiefs of police

**Figure 1:**
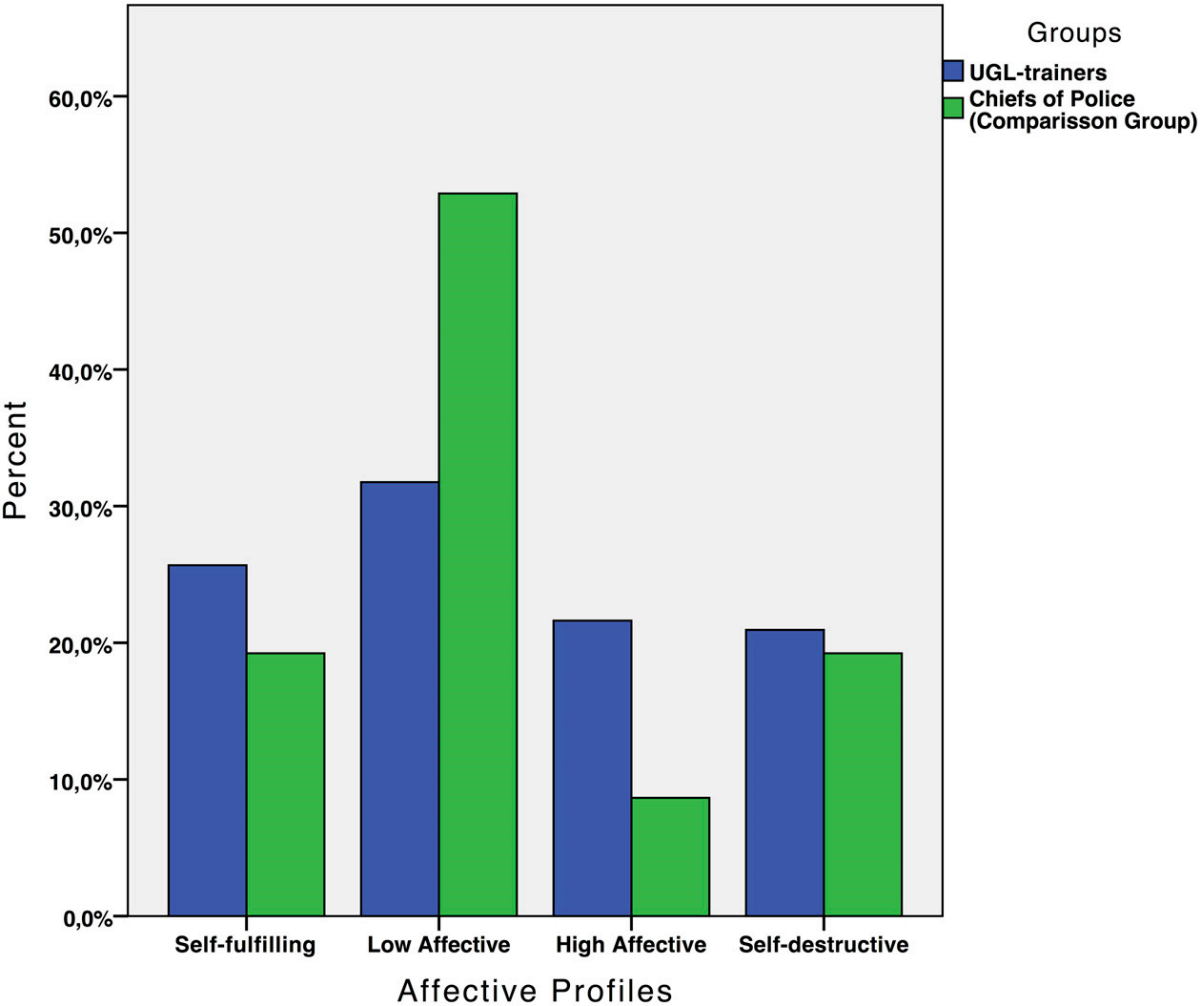
**Distribution of the affective profiles, self-fulfilling, high affective, low affective, and self-destructive, among UGL-trainers and the comparison group**.

The χ^2^-test showed a significant difference in the distribution of profiles among certified UGL-trainers and the comparison group: χ^2^(3, *N* = 252) = 14.24, *p* < 0.01. As expected, the self-fulfilling profile was more frequent among the certified UGL-trainers (25.7%) compared to the comparison group (19.2%). Nevertheless, among the UGL-trainers the low-affective profile was the most common (31.8%), followed by the self-fulfilling profile (25.7%), the high-affective profile (21.6%) and the self-destructive profile (20.9%). In the comparison group the distribution was as follows: low-affective = 52.9%, self-destructive = 19.2%, self-fulfilling = 19.2%, and high-affective = 8.7% (see Figure 1).

The low affective group, the most common profile in the whole sample, was used in the MLR analysis as the baseline group to test whether being a certified UGL-trainer or a Chief of Police (i.e., the comparison group) increased/decreased the odds of different combinations of PA and NA (i.e., type of affective profile). The results showed that (i) certified UGL-trainers compared to the Chiefs of Police were more likely to have a self-fulling profile than a low affective profile (OR = 2.22, *p* < 0.05), and that (ii) certified UGL-trainers compared to the Chiefs of police were also more likely to express a high affective profile than a low affective profile (OR = 1.43, *p* < 0.001).

### Differences in optimism, self-esteem, and locus of control

The correlation coefficients between the dependent variables (optimism, self-esteem, and locus of control) in the MANOVA varied between 0.15 and 0.57, which is below the 0.90 recommendations by Tabachnick and Fidell (2007) for performing a MANOVA. The MANOVA showed a significant effect of the affective profiles on optimism, self-esteem, and internal and external locus of control: *F*_(12,371)_ = 5.92, *p* < 0.001, Wilks' Lambda = 0.63, η^2^= 0.14.

There was a significant effect of the affective profiles on optimism: *F*_(3,143)_ = 16.06, *p* < 0.001, η^2^= 0.25. Bonferronis *post-hoc* test showed that UGL-trainers with a self-fulfilling profile scored higher (*p* < 0.001) in optimism (*M* = 3.3, *SD* = 0.3) compared to UGL-trainers with a self-destructive profile (*M* = 2.6, *SD* = 0.6). No differences (*p* = 0.09) were found between UGL-trainers with a self-fulfilling profile and those with a low-affective profile (*M* = 3.1, *SD* = 0.4) or between self-fulfilling and high-affective profiles (*M* = 3.1, *SD* = 0.4; *p* = 0.37). Moreover, both the low affective and high affective profile scored higher in optimism compared to the self-destructive profile (*p* < 0.001) see Table 2. Levene's test showed that the groups were not homogeneous: *F*_(3,143)_ = 4.95, *p* < 0.01, but Kruskall-Wallis non-parametric test showed a significant result anyway: χ^2^(3, *N* = 147) = 31.80, *p* < 0.001.

**Table 2:** **The means and standard deviation (in parentheses) in optimism, self-esteem, and internal and external locus of control for the four affective profiles among UGL-trainers**.

**Affective profile**	**Optimism**	**Self-esteem**	**Internal locus of control**	**External locus of control**	**Positive affect**	**Negative affect**
Self-fulfilling	3.3 (0.3)^↑^	3.6 (0.2)^↑^	3.3 (0.7)	1.9 (0.6)^↓^	4.3 (0.3)	1.3 (0.2)
Low affective	3.1 (0.4)^↑^	3.5 (0.3)^↑^	3.4 (0.6)	2.2 (0.5)	3.4 (0.6)	1.4 (0.2)
High affective	3.1 (0.4)^↑^	3.5 (0.3)^↑^	3.4 (0.7)	2.2 (0.6)	4.4 (0.3)	2.2 (0.4)
Self-destructive	2.6 (0.6)	3.1 (0.4)	3.1 (0.5)	2.4 (0.6)	3.4 (0.4)	2.2 (0.4)

↑ significantly (p < 0.001) higher than the self-destructive profile;

↓ *significantly (p < 0.001) lower that the self-destructive profile*.

The affective profiles differed in self-esteem as well: *F*_(3,143)_ = 20.35, *p* < 0.001, η^2^ = 0.30. Bonferronis *post-hoc* test showed that UGL-trainers with a self-fulfilling profile (*M* = 3.6, *SD* = 0.2) scored higher (*p* < 0.001) in self-esteem compared to UGL-trainers with a self-destructive profile (*M* = 3.1, *SD* = 0.4), but not compared to UGL-trainers with a low-affective profile (*M* = 3.5, *SD* = 0.3; *p* = 0.18) or compared to UGL-trainers with a high-affective profile (*M* = 3.5, *SD* = 0.3; *p* = 0.26). Moreover, both the low and high affective profile scored higher in self-esteem compared to the self-destructive profile (*p* < 0.001), see Table 2. Levene's test showed that the groups were not homogenous: *F*_(3,143)_ = 5.09, *p* < 0.01, but Kruskall-Wallis non-parametric test showed a significant result: χ^2^(3, *N* = 147) = 38.07, *p* < 0.001.

Finally, there was no significant effect of the affective profiles on internal locus of control: *F*_(3, 143)_ = 1.61, *p* = 0.19. However, there was a significant effect on external locus of control: *F*_(3, 143)_ = 3.89, *p* = 0.01, η^2^ = 0.08. Bonferronis *post-hoc* test showed that UGL-trainers with a self-fulfilling profile (*M* = 1.9, *s* = 0.6) scored lower (*p* < 0.01), in external locus of control compared to UGL-trainers with a self-destructive profile (*M* = 2.4, *s* = 0.6), but not different compared those with a low-affective profile (*M* = 2.2, *s* = 0.5; *p* = 0.38) or compared to UGL-trainers with a high-affective profile (*M* = 2.2, *s* = 0.6; *p* = 0.38) see Table 2. Levene's test showed that the groups were homogeneous regarding internal [*F*_(3, 143)_ = 2.06, *p* = 0.109] as well as external locus of control [*F*_(3, 143)_ = 0.83, *p* = 0.48].

## Discussion

The general aim of the present study was to examine personal predispositions of the certified UGL-trainers that might be important for leading UGL-courses and educating trainees. These courses have indeed focused on increasing self-awareness, self-esteem, optimism, internal rather than external locus of control, communication skills, and in giving opportunities for self-development, leadership, and empowerment. Specifically, we aimed to (1) compare profiles between UGL-trainers and managers/supervisors and to (2) investigate differences in personal characteristics among UGL-trainers using the affective profile model as the backdrop of the analyses.

We found that the low affective profile was the most common profile among the certified UGL-trainers. This finding was paradoxical since one characteristic of an individual with a low-affective profile is their low level of personal development (Garcia et al., 2014) and thriving (Norlander et al., 2005), while the UGL-trainers are expected to focus upon self-development, both as coach and personally. In order to become trainers, the UGL-trainers undergo several significant periods of self-development, which implies that the self-fulfilling profile should be expected to have been more markedly represented among them. The results found here might represent some type of stagnation within the UGL-trainers professional role or personal development. In other words, a “peak-out,” or having “peak-out” and perhaps a lack of intrinsic motivation. Alternatively, the sample of UGL-trainers used here might consist of a number of insufficiently coached trainers. This suggested lack of self-coaching, however, might only mirror the UGL-trainers' own situation in which they have all their attention to their trainees without opportunities for continued self-development. Whatever the reason, low affectivity has important links to motivation.

The low-affective profile is defined by a low level expression of both PA and NA. PA is characterized by feelings of commitment, enthusiasm, activity, energy whereas NA is characterized by feelings of fear, anger, guilt, and anxiety (Watson et al., 1988). Motivation is a prerequisite for initiating personal motivation and driving forward (Bandura, 1999). According to the tenets of self-determination theory, personal development is associated intimately with intrinsic motivation (Deci and Ryan, 2008) and low affectivity is linked to low levels of self-determination (Archer et al., 2008). Again, these findings imply some form of “stagnation” effect, suggesting the necessity of further investigation of long-life learning among UGL-trainers.

Nevertheless, according to the expectations the self-fulfilling profile was found to a greater extent among the UGL-trainers than in the comparison group. Specifically, (i) certified UGL-trainers compared to the Chiefs of Police were more likely to express a self-fulling profile than a low affective profile, and (ii) certified UGL-trainers compared to the Chiefs of Police were also more likely to express a high affective profile than a low affective profile thereby confirming the first hypothesis. Moreover, as indicated in the results, high PA profiles (i.e., self-fulfilling and high affective) and low NA profiles (i.e., self-fulfilling and low affective) reported higher self-esteem and optimism; but only the experience of high in PA and low in NA (i.e., self-fulfilling) was associated to low external locus of control. Accordingly, Archer et al. (2008) described the self-fulfilling profile as individuals with high degree of optimism, self-esteem, and internal locus of control. Garcia et al. (2014) claimed that individuals with a self-fulfilling profile have good personal relationships, accept themselves, masters their environment, have a high degree of personal development, perceive harmony, and have a sense of meaning in life. Certainly, the link between optimism and the self-fulfilling profile are well-established (Scheier and Carver, 1985; Garcia et al., 2014), as well as the link between PA and self-esteem (Ozyesil, 2012). Moreover, both self-esteem and PA are negatively associated with stress (Nima et al., 2013). In line with the findings presented here, a study of 150 executives at an automobile company showed that participants expressing high emotional intelligence and internal locus of control scored significantly higher on PA and scored significantly lower on NA (Kulshrestha and Sen, 2006; see also Hodges and Winstanley, 2012).

### Limitations and final remarks

First of all, the low Cronbach's alpha (0.59) for internal locus of control was below Nunnallys (1978) original reliability of 0.70. The reliability of external locus of control was even lower (0.47). Together with the attrition rate, these low values undermine the generizability of the present results. Secondly, the participation was undesirably low, 37%, and therefore representatively weak. Nevertheless, this is a common phenomenon of web-based as opposed to paper-based questionnaires (Shih and Fan, 2008). Thirdly, the categorization of affective profiles from PANAS responses has been critizied previously (Schütz et al., 2013). Nevertheless, the affective profiles derived through cluster analysis have produced the same affective profiles as applied here (MacDonald and Kormi-Nouri, 2013). Also in this line, it could be argued that the experienced sampling method (i.e., gathering online experience of emotions) should be more appropriate to study affective experience. Although, we see this as an exciting development in the construction of affective profiles it is important to notice that affective experience using experience sampling and retrospective reports differ from each other in important ways. Scollon et al. (2009), for instance, concluded that retrospective reports of affect seem to involve a dynamic process incorporating cultural information in the recollection of PA and NA, while on-line emotions are more strongly related to temperamental dispositions (see also Cloninger and Garcia, 2014).

Finally, although many governmental institutions in Sweden actually undergo the UGL-training, having the comparison group constituted of Swedish Chiefs of Police might limit the generalizability of the results presented here. In other words, the finding suggesting that the self-fulfilling profile was more common among UGL-trainers than among Swedish Chiefs of Police might actually reflect the actual profession. Other researchers have indeed found differences in temperament and personality profiles among physicians, lawyers, managers/executives, industrialists, architects, journalists, and artists (Akiskal et al., 2004). Physiscians, for example, showed a “warmer” temperament than most of the lawyers (Akiskal et al., 2004). Lawyers are probably the profession that is more alike the Chiefs of Police in our comparison group. Nevertheless, the professions in the Akiskal and colleagues' study overlapped in most of the attributes (Akiskal et al., 2004). For instance, Akiskal et al. (2004) described that a majority of both managers/executives and lawyers could be described as unremarkable in temperament and even phlegmatic. The phlegmatic temperament is described as inward and private, thoughtful, reasonable, calm, and patient (Eysenck, 1967). This definition might as well apply to the low affective profile (see Cloninger and Garcia, 2014), which for instance was the most common of the profiles among both UGL-trainers and Chiefs of police. Low affectivity, in comparison to self-fulfillment (i.e., high PA and low NA), leads to low performance during stress (Norlander et al., 2002), low degree of personal development and purpose in life (Garcia et al., 2014), and to low resilience (Norlander et al., 2005). In this line of thinking, our findings might show that there is an actual effect of being an UGL-trainer—the probability of self-fulfillment rather than low affectivity.

The present study offers the first analysis (for some criticism toward the UGL-training see Fellinger, 2012a,b,c; SNDC, 2013) of UGL-trainers' affectivity and personal attributes that might be markers of self-awareness, maturity, and adaptive coping skills (Garcia, 2011). Although our findings suggest that UGL-trainers are more likely to develop a self-fulfilling profile with high levels of optimism and self-esteem and low levels of external locus of control, seeing that the most common profile among UGL-trainers was the low affective it is then plausible to suggest that no efforts should be spared to continue the work improving the UGL-trainers' level of self-awareness. Certifications may need to eventually be refreshed. Continuous coaching by professional coaches in other areas or peer-coaching may be a solution. Professional psychologists, for instance, often undergo a life-long process of continuous counseling, hence, it might be a good idea to introduce similar dynamics among UGL-trainers.

“If you are pleased with what you are,

you have stopped already.

If you say, “It is enough,” you are lost.

Keep on walking, moving forward, trying for the goal.”

St. Augustine

### Conflict of interest statement

The authors declare that the research was conducted in the absence of any commercial or financial relationships that could be construed as a potential conflict of interest.
